# Development and validation of 'AutoRIF': software for the automated analysis of radiation-induced foci

**DOI:** 10.1186/2041-9414-3-1

**Published:** 2012-01-26

**Authors:** Andrew McVean, Simon Kent, Alexei Bakanov, Tom Hobbs, Rhona Anderson

**Affiliations:** 1 Centre for Cell Chromosome Biology, Division of Biosciences, Brunel University, Uxbridge UB8 3PH, UK; 2 Centre for Infection, Immunity and Disease Mechanisms, Division of Biosciences, Brunel University, Uxbridge UB8 3PH, UK; 3 Centre for Information and Knowledge Management, School for Information Systems, Computing and Mathematics, Brunel University, Uxbridge UB8 3PH, UK

## Abstract

**Background:**

The quantification of radiation-induced foci (RIF) to investigate the induction and subsequent repair of DNA double strands breaks is now commonplace. Over the last decade systems specific for the automatic quantification of RIF have been developed for this purpose, however to ask more mechanistic questions on the spatio-temporal aspects of RIF, an automated RIF analysis platform that also quantifies RIF size/volume and relative three-dimensional (3D) distribution of RIF within individual nuclei, is required.

**Results:**

A java-based image analysis system has been developed (AutoRIF) that quantifies the number, size/volume and relative nuclear locations of RIF within 3D nuclear volumes. Our approach identifies nuclei using the dynamic Otsu threshold and RIF by enhanced Laplacian filtering and maximum entropy thresholding steps and, has an application 'batch optimisation' process to ensure reproducible quantification of RIF. AutoRIF was validated by comparing output against manual quantification of the same 2D and 3D image stacks with results showing excellent concordance over a whole range of sample time points (and therefore range of total RIF/nucleus) after low-LET radiation exposure.

**Conclusions:**

This high-throughput automated RIF analysis system generates data with greater depth of information and reproducibility than that which can be achieved manually and may contribute toward the standardisation of RIF analysis. In particular, AutoRIF is a powerful tool for studying spatio-temporal relationships of RIF using a range of DNA damage response markers and can be run independently of other software, enabling most personal computers to perform image analysis. Future considerations for AutoRIF will likely include more complex algorithms that enable multiplex analysis for increasing combinations of cellular markers.

## Introduction

The use of markers, such as the phosphorylated variant of histone 2A (γ-H2AX), for the detection of radiation-induced double strand breaks (DSBs) is now standard practice for investigating biologically relevant doses of radiation (for a review of the field see Lobrich *et al *2010 [1]). For instance, the induction and subsequent repair kinetics of DSB has been determined in a range of cell types upon exposure to varying qualities and radiation doses [2-6]. Additionally, the development of antibodies specific for other relevant proteins in the DNA damage response (DDR) pathway have revolutionised our ability to investigate mechanistic aspects of DSB processing in interphase nuclei. For example, 53BP1 has been shown to localise to sites of DSB [7-9] and studies have implicated the localisation and retention of 53BP1 to be γ-H2AX dependant [10], Mdc1 dependant [11-13] and as a function of chromatin accessibility [14]. Compared to γ-H2AX, 53BP1 has a significantly improved signal to noise ratio and additionally has been applied for live cell visualisation of DSBs through generation of GFP fusion proteins [11,13,15,16]. Radiation-induced foci (RIF) assays are now being applied to assess clinical outcome to radiation [17] as well as evaluating risk complications associated with radiotherapies and diagnostics [18-20]. RIF are also being exploited as dosimetry biomarkers to identify individuals exposed to unknown levels of radiation [21] and for the assessment of cellular sensitivity, which could be utilised as to screen patients prior to radiotherapy or diagnostics [22].

To reliably quantify RIF there must be a clear definition of what actually constitutes a 'focus'. A RIF can be described as a peak of signal intensity, distinct from the background and therefore displaying a strong signal to noise (S:N) ratio. Factors that can reduce this S:N ratio include poor immunofluorescence staining as a consequence of the antibody employed or poor technique, auto-fluorescence within the sample, the number and distribution of RIF (e.g whereby individual RIF overlap) and the optical system used. Counting of RIF has typically been achieved by manual analysis through the optical binoculars of a fluorescence microscope or by taking images and counting the RIF on a digital screen [23-27]. If the latter, then additional factors which may influence the quantification of RIF include (1) characteristics of the objective lens, (2) the camera used to acquire the images, and (3) the way in which the images are subsequently analysed. Thus, the type of image acquisition and the numerical aperture of the objective lens used will directly affect the resolution of RIF, potentially impacting on the number of RIF counted. For instance, if a single image of one focal plane is acquired using a high numerical aperture (N.A. 1.4) lens, then the resulting narrow field of view will mean only a small proportion of the nucleus is actually sampled. This can be resolved by capturing images from multiple focal planes for subsequent analysis as sequential 2D images or as collapsed maximum intensity projections (MIP). The advantage of analysing from an MIP compared to sequential 2D slices are improved S:N, which is particularly useful when manually analysing from digital images, however overlapping RIF from separate focal planes will be visualised as the same single RIF potentially leading to inaccuracies when nuclei contain large numbers of RIF crossing multiple focal planes. With regards to the choice of camera, the ability to resolve RIF will be determined by the resolution of digital images acquired and therefore the sensitivity and range (bit depth) of the camera employed. Accordingly, a minimum of an 8-bit camera is essential to ensure optimal acquisition of all RIF. Regardless of any technological variations for image acquisition however, the principle limiting factor to achieve high-throughput, reproducible and accurate RIF quantification are the difficulties in manually discriminating between background and RIF, particularly when RIF lie on multiple focal planes, and which ultimately lead to varying results between operators and between labs.

A number of automated systems that lend themselves for the quantification of RIF are available commercially. Image analysis packages such as Imaris, ImageJ and CellProfiler, typically rely upon the application of an intensity threshold and minimum size parameters to isolate RIF from the background. Over the last decade systems specific for the automatic quantification of RIF have been developed [27-34]. The majority of these analysis solutions use a single 2D image to count foci, most commonly generated from the MIP of an image stack, but more recently efforts have been made to score multiple focal planes independently. Increasing the axial resolution, i.e. through the nuclear depth, is becoming more important as we ask more mechanistic questions on the spatio-temporal aspects of RIF such as determining the composition of RIF at varying times after irradiation and also, assessing the relevance of RIF in the formation of chromosome exchanges. Thus our aim was to develop a rapid, high-throughput, high fidelity automated RIF analysis platform that required minimum user input for the quantification of the number, size/volume and relative 3D distribution of RIF within individual nuclei.

## Material and methods

### Cell culture, irradiation and immunofluorescence

Primary human bronchial epithelial (NHBE) cells (Lonza) were cultured in complete medium (Lonza BulletKit CC-3170) which consists of bronchial epithelial basal medium supplemented with bovine pituitary extract (0.2%), insulin (0.1%), hydrocortisone (0.1%), gentamicin sulfate and amphotericin-b (0.1%), retinoic acid (0.1%), transferrin (0.1%), epinephrine (0.1%) and human epithelial growth factor (0.1%). Cells were seeded at a density of 3.5 × 10^3 ^cells/cm^2 ^and routinely sub-cultured at~ 80-90% confluence by trypsinising according to the suppliers guide. In brief, the cell sheet was rinsed with pre-warmed HEPES-BSS, incubated with Trypsin/EDTA solution at 37°C until > 90% of cells had rounded up and become detached before the addition of an excess of Trypsin Neutralisation Solution (TNS). For experimentation passage 3-7 NHBE cells were seeded onto sterilised glass microscope slides (Menzel) in quadriPERM^® ^dishes (Sigma) and cultured for between 2 and 3 days until 90% confluent. Cells were then exposed to ^60^Cobalt γ-rays (~0.33 Gy/min) at 37°C and incubated for varying lengths of time before being washed three times with ice-cold PBS and fixed in 4% paraformaldehyde at room temperature (RT) for 5-10 min. For immunofluorescence, cells were washed three times for 3-5 min each in PBS, permeabilised (5% saponin (w/v) and 5% triton X-100 (v/v) in PBS) for 20 min at RT and incubated in blocking buffer (5% foetal bovine serum in PBS) for 1 hr at RT. Cells were then incubated with primary antibody (mouse monoclonal anti-human 53BP1 (BD Biosciences Clone 19) 1:200 in blocking buffer) for 1 hr at RT, washed three times in PBS for 5 min each with agitation then incubated with secondary antibody (goat anti mouse IgG conjugated with AlexaFluor 555 (Invitrogen)) again for 1 hr at RT. Cells were subsequently washed three times for 5 min each in PBS with agitation, mounted and counterstained with vectashield containing 4',6-diamidino-2-phenylindole (DAPI).

### Image acquisition using widefield fluorescence microscopy

Images of fixed NHBE nuclei were acquired on an Axiovert 200 M microscope (Carl Zeiss) equipped with 100× NA 1.3 objective lens and Axiocam HR (14 bit) camera, and controlled with Axiovision software. Image dimensions of 1300 × 1030 at a pixel scale of 0.07 μm/pixel provided a field of view (FOV) of approximately 90 × 70 μm. The exposure time for each fluorescence channel was determined by examining multiple FOV for each slide to identify the highest intensity as to ensure images were not overexposed, whilst balancing the highest signal to noise ratio with the need to preserve the fluorescence signal across the whole slide. This was typically in the range of 100-600 ms for radiation-induced foci (RIF) and 30-80 ms for DAPI. Image stacks of 22-25 slices were acquired at 0.5 μm intervals from the central focal plane with FOV being selected randomly using the DAPI fluorescence channel ensuring every image stack contained at least one nucleus. Acquisition times varied but typically took in the region of 2 min/FOV. For subsequent automated analysis, each image stack was saved within its own folder as tagged image file format (TIFF) series.

### Image processing for analysis of radiation-induced foci (RIF)

#### Manual analysis

Manual analysis for the quantification of RIF was conducted on coded slides using Axiovision software (Carl Zeiss) to view the images. To categorise RIF into size integers an acetate sheet with two scaled circles corresponding to 0.5 μm and 1.0 μm was used. RIF smaller than the 0.5 μm circle were categorised as "small", RIF larger than this but smaller than the 1.0 μm circle were categorised as "medium" and any RIF larger than 1.0 μm in diameter were categorised as "large". RIF from multiple focal planes were counted and categorised according to size by scrolling up and down through the image stack, whereby the most in-focus area was used to determine the foci size (diameter). Where RIF had indiscriminate edges or were not sharp enough to discern a boundary and therefore a categorisation of size, they were classified as being "out-of-focus" (OOF).

#### Development of automated analysis system (AutoRIF)

**Figure 1:**
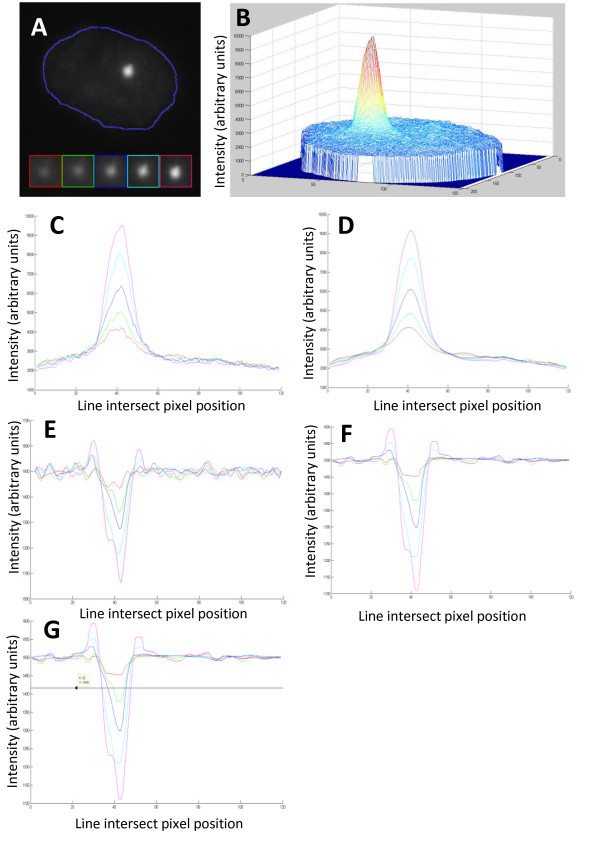
**Processing steps for identification of RIF**. (A) A single slice (2D) grey-scale image showing a nucleus, blue dashed line, with a single RIF. The RIF has been highlighted to show its appearance in subsequent slices and are colour-coded to C-G. (B) The same image is shown as a 3D intensity mesh and is also shown (C) for a single axis with coloured lines for the most central focal plane (n = magenta) and the 4 focal planes at 0.5 μm intervals beneath. (D) The same intensity mesh after filtering shows reduced noise. (E) A laplacian operator is applied to identify boundaries and a normalisation value of 1500 is added to ensure RIF values do not fall below zero. (F) A final filtering step removes noise before finally (G) a maximum entropy threshold is applied to identify RIF.

The approach taken to identify nuclei (DAPI positive) was based on Otsu's method of automatic image thresholding [35] whereby a binary mask for each acquired image is created that effectively removes all information from outside the nucleus (DAPI negative). A modified version of the sequential region labelling algorithm assigns x/y positional data for each nucleus within the image where the bounding rectangle in each image stack is used to crop the nucleus. Any nuclei smaller than one thousand pixels or lying on the boundary of an image are excluded. Image stacks of individual nuclei are then processed for the identification of RIF (AF 555 positive). This is achieved by taking each 2D grayscale image from a nucleus stack (Figure 1A, B) and applying a series of filters (Figure 1C-G) in order to arrive at the 2D parameters for each RIF present. These 2D parameters are further combined to achieve 3D reconstructions of the RIFs.

The filtering stage comprises several steps. First, a modified Hessian operator is applied to remove high-frequency noise (Figure 1D) which gives the effect of smoothing the plots to allow accurate RIF dimensions to be extracted. This step does not affect the mask outline that is made from the DAPI channel but does remove the noise spikes. Next, the Laplacian transform operator converts the smoothed image such that intensity peaks are translated into troughs or concavities (Figure 1E). Although there are fluctuations in curvature of the plot these can easily be distinguished from the RIF. Any noise which escapes the smoothing phase would have been amplified by the application of the Laplacian operator. Therefore, to remove any artificially enhanced noise from the 'Laplacian' image, the Crimmins filter is used. This brightens pixels that are significantly darker than their neighbours, whilst it darkens those that are lighter than their neighbours (Figure 1F). The maximum entropy threshold, derived from the data of the whole image stack, is then used to identify the RIF. The resulting binary image then undergoes the morphological closing operation, using a kernel size of 3x3, which has the effect of 'filling the gaps' in the individual foci (Figure 1G).

**Figure 2:**
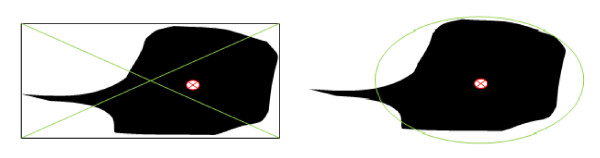
**Bounding of RIF to determine centre**. Example of irregular shaped RIF with a bounding rectangle (left) and ellipse (right). The 'true' centre of the RIF in both cases is shown by a red cross.

In the main, the binary RIF outlines are of irregular shape, therefore a simple approximation as a bounding rectangle is not sufficient to confidently determine RIF center (Figure 2 left). Elliptical approximation of a center at RIF's centroid (homogenous centre of mass) is a more robust approach (Figure 2 right). RIF centroid position and the parameters of the bounding ellipse are calculated from 'moments' of the binary region.

The concept of 'moments' originally comes from physics and statistics but can also be applied to the pixel distribution of the gray-scale image. The moment of the order p, q for a binary region is defined by:

$$m_{pq}=\Sigma_{\left(u,v\right)\in R^{u^{p}v^{q}}},$$

Where R is a set of region pixels for which the moment is calculated. The area of that region can be expressed as the zero order moment, described by:

$$\begin{array}{c} \text{A}\left(\text{R}\right)=|\text{R}|=\Sigma_{\left(u,v\right)\in R}1\\ =\Sigma_{\left(u,v\right)\in R^{u^{0}v^{0}}}=m_{00}\left(R\right) \end{array}$$

Coordinates of the gravity centre of the spot region (centroid) can be found by:

$$\overset{-}{x}=\frac{m_{10}\left(R\right)}{m_{00}\left(R\right)}=\frac{1}{\left|R\right|}\Sigma_{\left(u,v\right)\in R^{u^{1}v^{0}}}$$

$$\overset{-}{y}=\frac{m_{01}\left(R\right)}{m_{00}\left(R\right)}=\frac{1}{\left|R\right|}\Sigma_{\left(u,v\right)\in R^{u^{0}v^{1}}}$$

In order to calculate parameters that are position independent, such as rotation angle and ellipse eccentricity, the notion of central moments is used. The central moments are similar to the ordinary moments, except that they are calculated around the region Centroid $\left(\overset{-}{x},\overset{-}{y}\right)$:

$$\mu_{pq}\left(R\right)=\Sigma_{\left(u,v\right)\in R}{\left(u-\overset{-}{x}\right)}^{p}{\left(v-\overset{-}{y}\right)}^{q}$$

**Figure 3:**
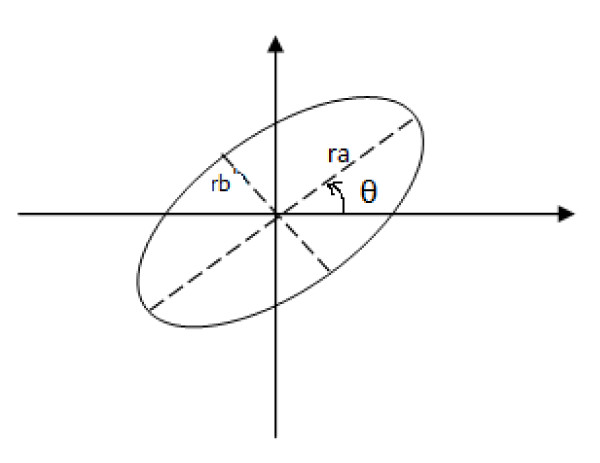
**Ellipse with rotation angle θ, major and minor radii *r_a _*and *r_b_***.

The rotation angle of the ellipse is the angle between x-axis and major ellipse axis (Figure 3). It is expressed via central moments of different orders described by:

$$\theta_{R}=\frac{1}{2}tan^{-1}\left(\frac{2*\mu_{11}\left(R\right)}{\mu_{20}\left(R\right)-\mu_{02}\left(R\right)}\right)$$

Eccentricity of the ellipse, described by:

$$\begin{array}{c} \text{Ecc}\left(\text{R}\right)=\frac{a^{1}}{a^{2}}\\ =\frac{\mu_{20}+\mu_{02}+\sqrt{{\left(\mu_{20}-\mu_{02}\right)}^{2}+4*\mu_{11}^{2}}}{\mu_{20}+\mu_{02}-\sqrt{{\left(\mu_{20}-\mu_{02}\right)}^{2}+4*\mu_{11}^{2}}} \end{array}$$

where, *a*_1 _= 2*λ*_1_, *a*_2 _= 2*λ*_2_, are multiples of eigen values *λ*_1_, *λ*_2 _of the symmetric matrix

$$A=\left(\begin{array}{cc} \mu_{20} & \mu_{11}\\ \mu_{11} & \mu_{02} \end{array}\right)$$

The minor and major radii of the approximation ellipse can be expressed via its eccentricity:

$$r_{a}=2*{\left(\frac{\lambda_{1}}{\left|R\right|}\right)}^{1/2}={\left(\frac{2a_{1}}{\left|R\right|}\right)}^{1/2}$$

$$r_{b}=2*{\left(\frac{\lambda_{2}}{\left|R\right|}\right)}^{1/2}={\left(\frac{2a_{2}}{\left|R\right|}\right)}^{1/2}$$

This localisation algorithm uses the above to return the number of RIF in a slice, and for each RIF their centre coordinates, area, rotation angle and radii.

**Figure 4:**
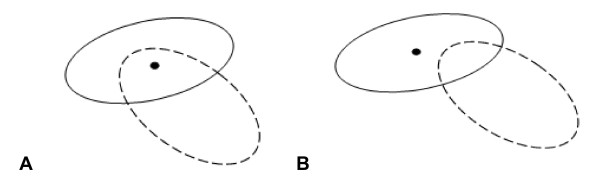
**Discrimination of overlapping RIF**. (A) Overlapping RIF will be categorised as a single RIF which spans multiple z-stacks since the centre (denoted by black spot) of the upper RIF lies within the area of the RIF below. (B) Overlapping RIF will be categorised as two individual RIF as the upper RIF lies outside the area of RIF below.

The 3D parameters of each RIF can be reconstructed from this by applying a custom algorithm that checks whether each RIF lies directly above or below each other in neighbouring slices in the image stack. For instance, if two RIF lie above each other at slices N and N+1 and the centre of any of the RIF lies within the area of the other focus, then these two RIF should be considered as a part of the whole in 3D. If the RIF lie above each other at slices N and N+1 and neither have its centre within the area of the other focus then these two RIF are considered independent of each other (Figure 4). Where RIF overlap but are separated by a z-slice with no RIF then they are also considered to be independent of each other. This 3D reconstruction process also keeps track of the weighted average of the z-coordinate for every RIF, together with × and y-coordinates. Since the weighted average utilises the same concept as the centroid, so the output of the procedure is a weighted 3D focus coordinate. This raw data is automatically inserted into Excel spreadsheets enabling further processing to categorise RIF by size, volume, intensity, 3D position within nuclei and also distance between each RIF within individual nuclei.

### Application of automated analysis system

**Figure 5:**
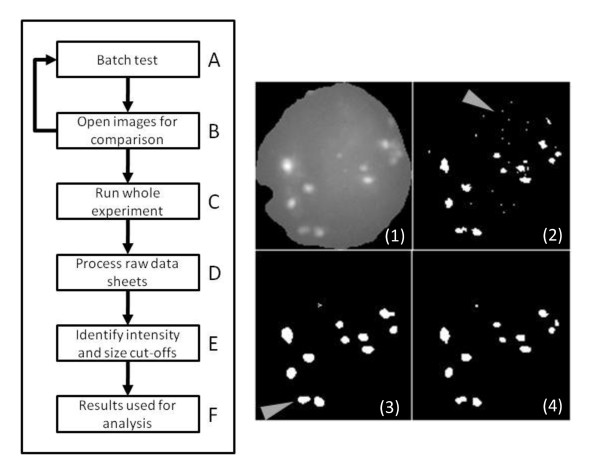
**Workflow for image processing and analysis**. Workflow A-F shows the process for analysing images in each experiment. Panel 1 is an example of a greyscale raw image with nucleus outlined and 53BP1 foci visible. Panel 2 shows a binary image with minimal thresholding and an arrow highlighting salt & pepper noise while panel 3 shows the effects of increasing filtering resulting in altered morphology of foci. Panel 4 is a compromise that generates a binary image that more closely resembles the raw image.

**Figure 6:**
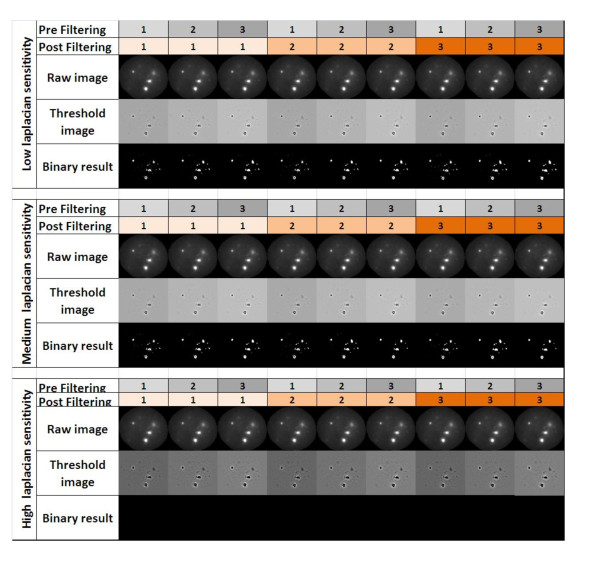
**Effects of RIF filtering steps**. Pre-laplacian, laplacian and post-laplacian filtering effects on an image of a single nucleus with 53BP1 foci. The optimal filtering combination for this nucleus would be 3 pre-filtering steps, medium sensitivity laplacian filter and 3 post-laplacian filtering steps. ~10% of nuclei are batch tested (covering test and sham slides) to identify the optimal combination of filters to be used for each experiment.

Figure 5 details the workflow performed for each experiment. Initially a sample of images is selected from each experiment and used to test the settings for the number of filtering iterations required (Figure 5A). Within this test batch the optimal settings may vary from image to image, therefore the final settings used for each was determined by the minimum number of filtering iterations that could be used without introducing artefacts. For instance, if too few filtering iterations were used, excessive noise termed as 'salt and pepper' (Figure 5 panel 2) will be seen. Increasing the pre-Laplacian filtering iterations dramatically reduces the noise and also smoothes the outline of foci to become more circular (Figure 5 panel 3). Post-Laplacian filtering has a limited effect of reducing the background noise but has less of an impact on the outline of foci. Alteration of the Laplacian sensitivity can also be tested however this had no effect on the images analysed in this study and so was not changed from its minimum setting. A comparison of the effects of the different filtering steps is shown in Figure 6. Once the settings have been established then the entire experiment is processed (Figure 5).

## Results

Primary human bronchial epithelial (NHBE) cells were irradiated with sham or 2 Gy ^60^Co-γ-rays (0.33 Gy/min) and fixed with 4% paraformaldehyde at varying times after exposure. The recruitment of 53BP1 proteins to DNA double strand breaks (DSB) were detected using mouse anti-human 53BP1 (Clone 19 BD Biosystems) antibodies and secondary goat anti-mouse IgG (Invitrogen) antibodies tagged with Alexafluor 555. To test and validate AutoRIF for the analysis of radiation-induced foci (RIF), digital images from a single focal plane (2D) and image stacks from multiple focal planes (3D) were acquired using a widefield microscope (100 nuclei per time point) and used for non-biased analysis using both manual and AutoRIF methods.

**Figure 7:**
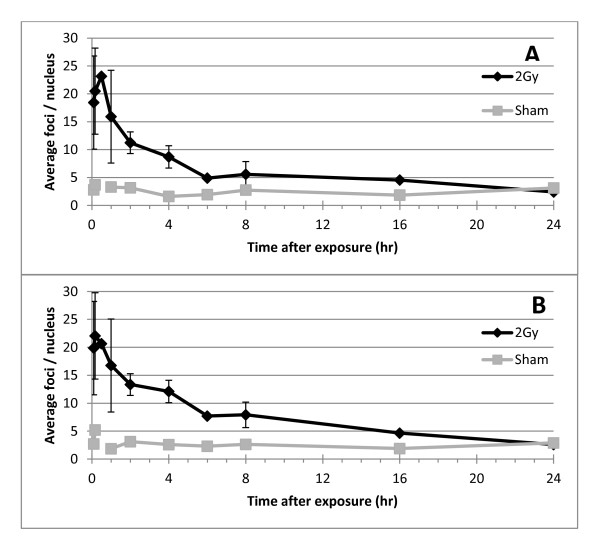
**Induction and persistence of 53BP1 foci in NHBE cells exposed to 2 Gy γ-rays**. The average number of 53BP1 foci/nucleus was quantified by (A) AutoRIF software and (B) manual analysis of the same 3D image stacks. Note the concordance between the two scoring methods. Averages are from multiple independent experiments (error bars represent standard deviation between independent experiments).

**Figure 8:**
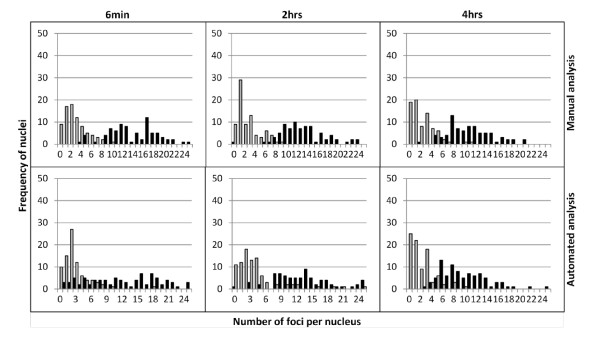
**Frequency distribution of 53BP1 foci/nucleus in NHBE cells 6 mins, 2 hrs and 4 hrs after exposure to 2 Gy γ-rays (black bars) or sham (grey bars), quantified by manual and AutoRIF analysis of the same 3D image stacks**.

**Figure 9:**
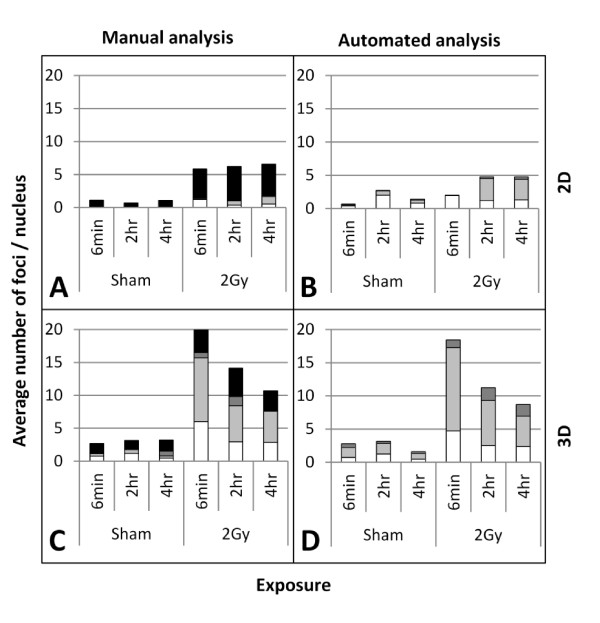
**Average number of 53BP1 foci/nucleus observed at different times after exposure to 2 Gy γ-rays categorised into three size integers;** < 0.5 μm (white), 0.5-1 μm (grey), > 1 μm (dark grey) and out-of-focus (black) scored by manual 2D and 3D analysis (A&C) and AutoRIF 2D and 3D analysis (B&D) of the same images. Averages are from at least two independent experiments.

As Figure 7 shows there is an excellent concordance between 3D manual and 3D AutoRIF quantification over a whole range of sample time points (and therefore range of total RIF/nucleus) after exposure to sham and 2 Gy γ-rays. The average number of 53BP1 foci at each time-point and the overall trend in induction and decline is consistent between both analysis methods demonstrating 3D AutoRIF detects RIF in nuclei with the same reproducibility to that of an experienced RIF analyst. This consistency was also observed when the trends in distribution of RIF in individual nuclei were compared (Figure 8). The major difference between manual and AutoRIF quantification was in the discrimination of what constituted a RIF (based on a minimum size and signal intensity) particularly in 2D images and, in the subsequent categorisation of RIF into different size integers (Figure 9). Essentially, since all RIF are categorised by AutoRIF we see a slightly larger proportion of medium and large sized 53BP1 foci/nucleus compared to that obtained manually i.e. no RIF is categorised as OOF by AutoRIF (Figure 9). The limited nuclear depth-of-field sampled in 2D compared to 3D image stacks is clearly reflected by a lower average number of RIF/nucleus when quantified by either method, however a greater correspondence between methods is achieved when 3D image stacks are quantified (Figure 9). For instance using the 2 hr time-point in Figure 9 as an example, 53BP1 foci were classified as comprising an average of 0.4/0.6/0 and 1.2/3.3/0.2 small/medium/large RIF/nucleus when analysed by 2D manual or AutoRIF methods respectively and, as an average of 2.9/5.5/0.8 and 2.5/6.7/2.0 small/medium/large RIF/nucleus when analysed by 3D manual and AutoRIF methods, respectively. Thus, 3D AutoRIF effectively enables the size (linear measurements as area or volume) of all RIF detected within the entire nuclear volume to be reliably quantified thereby providing valuable biologically-relevant data to be generated in a fraction of the analysis time.

**Figure 10:**
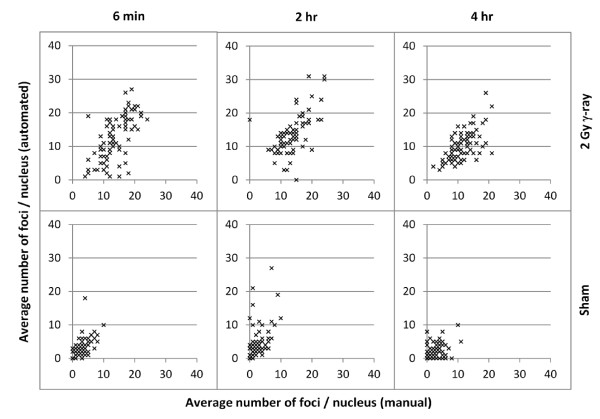
**Correlation plots comparing manual (x-axis) and AutoRIF (y-axis) analysis of RIF (100 nuclei per time-point) using the same 3D image stacks**. Comparisons were carried out for 6 min, 2 hr and 4 hr time-points.

To directly compare AutoRIF and manual quantification, correlation plots were generated for three different sample time-points giving coefficients of 0.63, 0.61 and 0.63 for 6 min, 2 hrs and 4 hrs respectively after 2 Gy exposure, and 0.61, 0.52 and 0.45 at 6 min, 2 hrs and 4 hrs after exposure to sham irradiation (Figure 10). Although no significant difference between the means derived from the two methods (p < 0.05) was found, outliers were identified for the purpose of reviewing the precise images involved. In all cases these were shown to represent either very closely overlapping nuclei or, poorly stained nuclei with excessive fluorescence background that could be misinterpreted as RIF. Thus, there is a need to ensure minimal inclusion of such image stacks in each data-set prior to AutoRIF processing.

## Discussion

The quantification and measurement of RIF is a rapidly developing field. The benefits and limitations of the RIF assay have been reviewed [36] but there has yet to be a standardisation in the method for RIF analysis. Of the automated systems currently available, the majority utilise filtering processes to enhance contrast between signal (RIF) and noise (background). The 'top hat' filter is commonly used in conjunction with smoothing filters to improve contrast for RIF analysis [34,37-40] which then allows a threshold to be applied to segment RIF from the background. The application of a fixed threshold alone is possible but there must be consistent contrast between RIF and background for this to be really effective [33]. It is more practical to use a dynamic threshold that changes automatically with each image based on the frequency distribution of pixel intensities (histogram) [41]. Alternatively, the identification of the centres of RIF from peaks of intensity (maxima) used in conjunction with water-shedding to identify the periphery of RIF [31] can be used. One issue with this is that it can lead to unnecessary segmentation of larger RIF and over-estimates from noisy background which although has been rectified with more complex algorithms [42], requires significantly more processing power and time.

The approach taken here was to identify nuclei using the dynamic Otsu threshold, which was then used as a mask to exclude non-nuclear signal from the RIF image. The Otsu method was applied to a MIP image produced by a projection of all stack images into a single one. Using such a composite image for nuclei segmentation reduces the chance of false-positives in Otsu methods when some of the images in each stack have only noise and no distinct nuclei. Image contrast of RIF was then enhanced using a Laplacian filter with smoothing processes, which reduces the risk of amplifying noise and generating artefacts. Finally, the maximum entropy threshold, based on the histogram of all the images in a stack (global), is applied to segment RIF from background in what has been described as pseudo 3D processing [34]. The rationale for including all images from the stack for the threshold calculation instead of calculating the threshold for each image separately is based on the following: noise is accumulated into the histogram from all the images and given that it is of the same average value across, becomes more distinct as a class. As well as noise, the signal (RIF) curvature spikes also get accumulated in the global histogram making the separation between noise and signal bigger. This makes the maximum entropy threshold class separation more precise. If the histogram were based on every individual image, in images with no distinct RIF, the maximum entropy method would segment the noise into two classes: strong and weak noise, so the stronger noise would be mistaken for RIF. However when the common threshold value based on the global histogram is applied to the whole stack, the images that do not have distinct RIF would be subject to the same threshold as used in images containing RIF. This prevents the noise from being exaggerated in slices without RIF. There is also the functionality to combine histograms from entire folders of results to further increase the strictness of the threshold, which extends the range of intensities that can be analysed within an experiment. The two filtering methods discussed (top hat and Laplacian) are not mutually exclusive, in fact a combination of Laplacian and top hat filtering has been used to identify similar objects in images from chest X-rays [43].

At each stage of this development validity checks using binary images and comparisons of overall scores were made, these checks appear to be standard for systems involving the quantification of foci [42], but detailed validation in relation to size is not [28]. Our results show there to be an excellent correlation between AutoRIF and manual analysis for the average number of RIF per nucleus, trends in induction and decline of RIF over time and, the frequency distributions for nuclei (Figures 7 and 8) for cells exposed to sham and 2 Gy radiation, while work is ongoing to assess these comparisons across a range of radiation doses. There is also good concordance between measurements of RIF size, but comparisons between manual and AutoRIF size proportions should be considered cautiously as the measurements are different. In the manual counting method, each RIF is only considered on a single focal plane and only the diameter is measured, for AutoRIF the volume is measured based on the theoretical 3D periphery of the RIF, (that is an elliptical approximation of the true boundary); the size integers applied to the AutoRIF score assume RIF to have an even and regular outline, which we know to be incorrect. A more accurate method for comparison between the methods would require more complex modelling of foci.

Some differences between manual and AutoRIF quantification were observed (Figure 7, 8, 9, 10) however these were identified to be due to outliers in the data caused by the very close bounding of two nuclei and poor fluorescence signal (data not shown). Thus, where AutoRIF is to be used in conjunction with images that were acquired using an automated image acquisition platform then it would be recommended to screen images e.g. as a thumbnail prior to the analysis processing. In addition to the above consideration, the application of any automated analysis system and subsequent interpretation of the data generated relies on the operators initial input of threshold parameters. This is no different for the application of AutoRIF whereby a robust regime for the experimental batch optimisation of filter settings, as described in Figures 5 and 6, is critical to ensure rigour and reproducibility. This filter optimisation process will be the same irrespective of dose or radiation quality meaning the software is flexible and applicable for a range of exposure types although it is likely that optimal parameters will differ for varying exposure types. For instance, exposure to high-LET radiation will (depending on the time after radiation) result in multiple, closely spaced RIF in those nuclei traversed meaning there is a potential for multiple small RIF to be identified as single larger RIF, if filter settings are not initially validated by comparing with the original raw images for that experiment. This batch optimisation process is by no means a caveat, indeed on-going developments of AutoRIF could incorporate a form of iterative or reinforcement machine learning, whereby the operator is presented with a sample image of what the algorithm believes is correct, the operator can then correct the image and the algorithm automatically adapts.

The main benefit of this analysis tool is the rapid turnaround of data. A single experiment with 10 slides and 100 nuclei per slide takes ~1-2 weeks to categorise all the RIF into size integers if manual analysis methods are used. Although reproducible this method is subject to bias and error. However, the same data can be obtained with more depth of information and consistency within 2 hrs (depending on hardware) with minimal operator input using AutoRIF. Specifically, consistent measurement of RIF size and signal intensity is generated for all RIF detected that is based on the number of pixels and is therefore continuous data rather than pre-defined categories. Further, this system generates x, y and z positional data for each RIF and provides distances between each RIF to all other RIF and also, to the nuclear centre, in each nucleus meaning that the spatial relationship of RIF can be studied at varying times after induction. Thus in addition to rapid quantification of RIF size (volume) and intensity, this automated system is a powerful tool for studying spatio-temporal relationships of RIF using a range of DDR markers.

## Competing interests

The authors declare that they have no competing interests.

## Authors' contributions

AB, SK and TH developed the analysis software with ADM and RA participating in its design. ADM performed all experiments and carried out the validation analysis. RA and ADM drafted the manuscript with contributions from TH and SK. All authors have read and approved the final manuscript.
